# A personalized biomimetic dual-drug delivery system via controlled release of PTH_1-34_ and simvastatin for *in situ* osteoporotic bone regeneration

**DOI:** 10.3389/fbioe.2024.1355019

**Published:** 2024-01-31

**Authors:** Tongtong Xu, Shang Gao, Nan Yang, Qi Zhao, Yutong Zhang, Tieshu Li, Zhihui Liu, Bing Han

**Affiliations:** ^1^ Department of Prosthodontics, Hospital of Stomatology, Jilin University, Changchun, China; ^2^ Jilin Provincial Key Laboratory of Tooth Development and Bone Remodeling, Jilin University, Changchun, Jilin, China; ^3^ Department of Stomatology, Fifth Affiliated Hospital of Sun Yat-Sen University, Zhuhai, Guangdong, China; ^4^ School of Pharmaceutical Sciences, Jilin University, Changchun, Jilin, China

**Keywords:** bone regeneration, osteoporosis, drug delivery system, PTH_1-34_, simvastatin

## Abstract

Patients with osteoporosis often encounter clinical challenges of poor healing after bone transplantation due to their diminished bone formation capacity. The use of bone substitutes containing bioactive factors that increase the number and differentiation of osteoblasts is a strategy to improve poor bone healing. In this study, we developed an *in situ* dual-drug delivery system containing the bone growth factors PTH_1-34_ and simvastatin to increase the number and differentiation of osteoblasts for osteoporotic bone regeneration. Our system exhibited ideal physical properties similar to those of natural bone and allowed for customizations in shape through a 3D-printed scaffold and GelMA. The composite system regulated the sustained release of PTH_1-34_ and simvastatin, and exhibited good biocompatibility. Cell studies revealed that the composite system reduced osteoblast death, and promoted expression of osteoblast differentiation markers. Additionally, by radiographic analysis and histological observation, the dual-drug composite system demonstrated promising bone regeneration outcomes in an osteoporotic skull defect model. In summary, this composite delivery system, comprising dual-drug administration, holds considerable potential for bone repair and may serve as a safe and efficacious therapeutic approach for addressing bone defects in patients with osteoporosis.

## 1 Introduction

Osteoporosis is one of the most widespread metabolic bone diseases worldwide. It has been reported that the number of individuals with osteoporosis exceeds 200 million, and this number is still rising in recent decades (Langdahl, 2021; Khandelwal and Lane, 2023). Osteoporosis is characterized by declined bone density, sparse bone trabeculae, and degenerative bone microarchitecture, which often lead to bone fractures and defects (Zhang H. et al., 2022). Bone substitutes or bone implants are often used for bone defects, but patients with osteoporosis face challenges in osseointegration due to reduced osteoblastic activity (Chandran and John, 2019). It is therefore crucial to consider these limitations when treating patients to achieve optimal outcomes.

In response to the difficulties posed by osteoporotic bone defects, a “Three in One” strategy has been proposed by researchers. The strategy includes proper bone grafting, anti-osteoporosis treatment, and subsequent bone regeneration promotion (Chen et al., 2022). Traditional bone grafts include autogenous bone, allogeneic bone, and xenografts, but supply constraints and potential immunological reactions remain substantial obstacles (Jin et al., 2021; Zhao et al., 2021). Advances in tissue engineering via biomaterial carriers have led to an opportunity to achieve tissue restoration and to deliver biological factors locally (Martin and Bettencourt, 2018; Wani et al., 2021). Methacrylate gelatin (GelMA), a hydrophilic polymeric network hydrogel, is a popular drug delivery vector and cell culture matrix due to its excellent physiological function, high water content, and controlled physicochemical properties (Sun et al., 2018; Wang F. et al., 2021). However, the mechanical characteristics of the hydrogels are significantly inferior to those exhibited by bone tissue, and thus, these hydrogels may provide inadequate support for subsequent bone tissue growth. Polylactic acid (PLA) is another degradable polymer which has received Food and Drug Administration (FDA) approval for osteoconductive scaffolds, and it is characterized by the exceptional mechanical strength similar to that of bone tissue (Barbeck et al., 2017; Grémare et al., 2018; Tilkin et al., 2020; Anita Lett et al., 2021; Xu et al., 2022). In addition, the 3D-printing properties of PLA make it particularly suitable for creating custom-fit scaffolds that match irregularly-shaped bone defects (Han et al., 2020).

Parathyroid hormone (PTH)_1-34_, a growth factor approved by the FDA as an anti-osteoporotic drug, is used to promote bone anabolism for the clinical treatment of osteoporosis (Hauser et al., 2021). Previous studies have suggested that PTH increases the number of osteoblasts by reducing apoptosis of these cells and exerts positive effects on bone (Silva and Bilezikian, 2015; Xie et al., 2017; Jiang et al., 2018; Chen et al., 2021; Rocha et al., 2021). Bone formation is the result of a rise in the quantity and differentiation of osteoblasts. As such, the administration of multiple bioactive factors may achieve the desired results within the skeletal environment (Wani et al., 2021). Simvastatin (SV) has demonstrated the ability to improve osteogenic differentiation by improving alveolar bone recovery, implant osseointegration, and bone tissue regeneration in various studies (Wang et al., 2018; Xu et al., 2018; Delan et al., 2020; Dang et al., 2021; Xu et al., 2021; Zhu et al., 2021). The systemic administration of PTH and SV produces a more significant osteogenic effect than either treatment alone (Jeon and Puleo, 2008; Tao et al., 2015; Tao et al., 2016). However, it is important to consider that systemic administration can result in high doses and low bioavailability, which may lead to potential toxicity and increased costs (Meng et al., 2022). Alternative approaches to drug administration may be needed to achieve more favorable outcomes.

**FIGURE 1:**
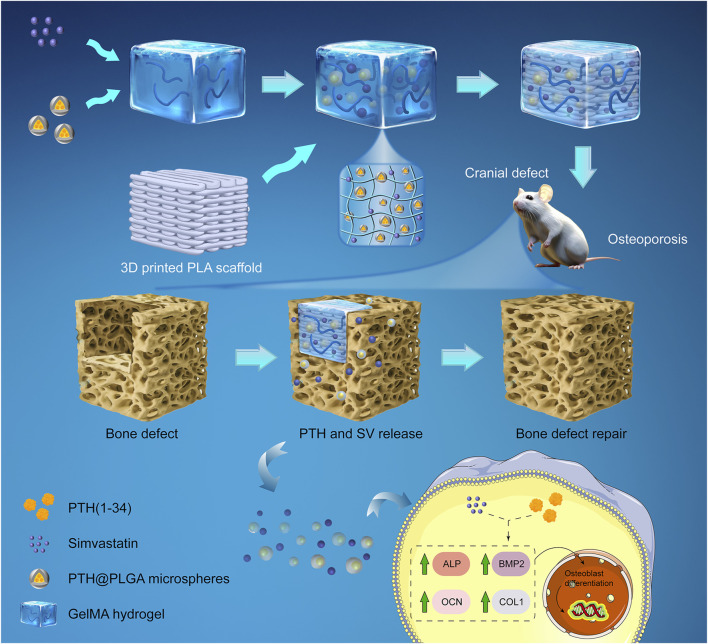
Schematic of a composite system for *in situ* osteoporotic bone repair. A dual-drug delivery system was composed of PTH@PLGA microspheres and SV, which were encapsulated in a GelMA hydrogel and enhanced by a 3D-printed PLA scaffold for compressive strength. The delivery system placed in the osteoporotic cranial defect controlled the release of PTH and SV, enhanced osteoblast marker (ALP, BMP2, COL1, OCN) expression, and improved bone regeneration.

The background provided indicates that a personalized dual delivery system consisting of bioactive PTH_1-34_ and SV should be considered when addressing osteoporotic bone defects. Therefore, we hypothesized that a composite PLA and GelMA scaffold loaded with PTH_1-34_ and SV to promote higher osteoblast numbers and differentiation could be employed as a bioactive bone scaffold to address osteoporotic bone defects (see Figure 1). In this study, a composite system composed of a 3D bioprinted PLA scaffold and GelMA was utilized to replicate the physical properties and personalized morphology of bone defects while concurrently controlling the release of osteoinductive factors. PTH_1-34_ was packed in poly (lactic-co-glycolic acid) (PLGA) microspheres to prevent early exposure to body fluids and to mitigate its short half-life. We assessed the osteogenic potential of the engineered delivery system in MC3T3-E1 cells *in vitro* as well as in osteoporotic rat cranial defects *in vivo*. Our results indicate that the dual release of SV and PTH_1-34_ from the delivery system upregulates osteogenic genes and proteins, thereby accelerating the healing process of osteoporotic bone defects. Overall, these findings highlight the potential effectiveness of the personalized biomimetic dual delivery system for osteoporotic bone repair.

## 2 Methods

### 2.1 Main reagents and materials

Medical-grade PLGA and PLA polymers were obtained from Daigang Bioengineering Co., Ltd. (Jinan, China). GelMA was obtained from Suzhou Yongqinquan Intelligent Equipment Co., Ltd. (Suzhou, China). SV was obtained from Macklin Biochemical Technology Co., Ltd. (Shanghai, China), and PTH_1-34_ was obtained from Psaitong Biotechnology Co. Ltd. (Beijing, China). The MC3T3-E1 cell line was a gift from Jilin University Stomatology Hospital. *qRT-PCR* related reagents were purchased from Yeasen Biotech (Shanghai, China). Antibodies were purchased from Proteintech or Servicebio Biotechnology Co., Ltd. (Wuhan, China). Other reagents were obtained from Solarbio (Beijing, China) or Beyotime (Shanghai, China).

### 2.2 Preparation of the PTH_1-34_/SV dual-drug delivery system

#### 2.2.1 PTH_1-34_@PLGA microspheres and encapsulation efficiency

A multiple emulsion method was used to prepare PTH_1-34_@PLGA microspheres as previously reported (Eswaramoorthy et al., 2012). Briefly, 500 μL of 100 μM PTH_1-34_ solution was emulsified in 5 mL dichloromethane PLGA solution, with a power of 15 W in an ice bath form a water-in-oil (w/o) emulsion. With vigorous stirring, the w/o emulsion was gradually added to 40 mL of 1% PVA aqueous solution to form a w/o/w solution. The solution was stirred overnight at room temperature to evaporate dichloromethane.

For the encapsulation efficiency, 10 mg PTH_1-34_@PLGA microspheres was added into a mixture of 0.9% NaCl and dichloromethane at room temperature to release PTH_1-34_ in the microspheres. The supernatant was collected by centrifugation to measure the concentration of PTH_1-34_. Encapsulation efficiency is determined by the following Formula (1):

$$\text{Encapsulation }\text{efficiency}\%=\frac{\text{Actual}\text{drug}\;\text{concentration}}{\text{Theoretical}\;\text{drug}\;\text{concentration}}\times 100\%,$$ (1)

#### 2.2.2 The PTH_1-34_/SV dual-drug delivery system

A 3D bioprinter (Sunp Biomaker Pro series, Sunp Biotech, Beijing, China) was employed to produce PLA scaffolds using a fused deposition technique. Cuboid models 10 mm × 10 mm in size were used for *in vitro* characterization and cell culture, while cylindrical models with a diameter of φ8 mm were used for animal models. A 10% (w/v) solution of GelMA and a 0.1% (w/v) solution of photoinitiator were dissolved according to the guidelines provided by the manufacturer, and then passed through a filter membrane. 0.4 mg PTH_1-34_@PLGA microspheres and 0.1 mg/mL SV were added into GelMA solution at 37°C, and the prepared mixture was crosslinked under 405 nm UV light after injection into the PLA scaffold. Finally, the dual-drug delivery system was obtained and kept at 4°C before being used.

### 2.3 Characterizations

#### 2.3.1 SEM

All samples were lyophilized and observed with a scanning electron microscope (JSM-IT500A) after gold spraying. The size of the microporous structure was measured using ImageJ.

#### 2.3.2 Porosity

Porosity was determined using the ethanol immersion method (Arumugam et al., 2020). Briefly, the initial mass of the dried samples was first measured as *W*_*i*_, and then the sample was immersed in ethanol medium and vacuumed. The final weight was quickly measured and recorded as *W*_*e*_. The porosity was performed based on the following Formula (2):

$$\text{Porosity }\%=\frac{W_{e}\;-\;W_{i}}{V\times \rho \;}\times 100\%,$$ (2)

where *V* is the total volume of the sample, and *ρ* is the specific mass of ethanol at atmospheric temperature, 0.789 g/mL.

#### 2.3.3 Water absorption

The water absorption was measured based on the ASTM standard D570 (Alam et al., 2020). The initial weight of the sample was first recorded as *W*_*0*_, and then the sample was submerged in neutral PBS at 37°C (Wu et al., 2020). After a 24-h immersion period, the sample was removed from the PBS, and the weight was recorded as *W*_*1*_. The water absorption rate of the sample was performed according to the subsequent Formula (3):

$$\text{Absorption rate }\%=\frac{W_{1}\;-\;W_{0}}{W_{0}\;}\times 100\%,$$ (3)

#### 2.3.4 Degradability and *pH* value

The sample was weighed as W_0_ and then transferred into neutral PBS at 37°C. A pH meter (INESA, China) was used to measure the pH around the samples. The remaining weight of the sample at a predetermined time was *W*_*t*_. The weight loss was determined according to the subsequent Formula (4):

$$\text{Weight loss }\%=\frac{W_{0}\;-\;W_{t}}{W_{0}\;}\times 100\%,$$ (4)

#### 2.3.5 Mechanical properties

A universal mechanical testing machine (AG-XPLUS10KN, Shimadzu, Japan) was employed to test the mechanical properties. Compression properties were examined with a speed of 1 mm/min. Stress‒strain curves corresponding to strains of 0%–10% were used to calculate the compression modulus.

### 2.4 *In vitro* release of PTH_1-34_ and SV from the delivery system

The samples were immersed in neutral PBS and underwent incubation at a temperature of 37°C. The solution was obtained at the predetermined time, and the absorbance values at 238 nm (SV) and 280 nm (PTH_1-34_) were measured by a spectrophotometer (UV-2100, Unico, China) (Yuan et al., 2022). The cumulative drug release was plotted in a line graph.

### 2.5 Hemolytic tests

Blood hemocompatibility was assessed using methods described in previous studies (Ma et al., 2018). Samples incubated in PBS at 37°C, diluted with 1:1.25 fresh rat blood and re-incubated for an hour before centrifuged. Supernatant absorbance at 545 nm was measured. PBS and distilled water served as negative and positive controls, respectively. The hemolysis rate (HR) was determined according to the subsequent Formula (5):

$$\text{HR }\%=\frac{A_{t}-\;{A\;}_{nc}}{{A\;}_{pc}-\;{A\;}_{nc}}\times 100\%,$$ (5)

where *A*_*t*_, *A*_*nc*_, and *A*_*pc*_ denote the absorbances of test, negative, and positive controls.

### 2.6 *In vitro* cell experiments

#### 2.6.1 Cell viability assay

Cranial-derived preosteoblastic MC3T3-E1 cells were cultivated in an incubator consisting of a 37°C environment supplemented with 5% CO_2_. α-MEM containing 10% fetal bovine serum and 1% penicillin‒streptomycin was used. The cells were seeded in 96-well plates and cultured with PTH_1-34_ or SV containing culture medium. After incubation for 1, 3, and 5 days, 100 μL diluted CCK-8 solution was added, and OD at 450 nm was measured.

#### 2.6.2 Live/dead cell staining

MC3T3-E1 cells were cultivated in 24-well plates for 48 h, and the cells were visualized by calcein-AM (1:1000) and PI (1:500) according to the assay instructions. Images were obtained using fluorescence microscopy (Olympus, Japan). The percentage of live/dead cells was counted using ImageJ.

#### 2.6.3 Alkaline phosphatase (ALP) and Alizarin red (AR) staining

MC3T3-E1 cells were cultured in osteogenic medium containing 10 mM disodium β-glycerophosphate, 50 μg/ml L-ascorbic acid, and 10^−7^ M dexamethasone. Cells were fixed in 4% paraformaldehyde before ALP and AR staining performed. Calcium nodules were lysed in 10% (w/v) cetylpyridinium chloride for quantification, and the OD values were measured at 562 nm (Fang et al., 2016).

#### 2.6.4 Immunofluorescence staining

Sterilized samples were wetted with culture medium overnight and then seeded with a density of 1500 cells/mm^3^ (Han et al., 2020; Wu et al., 2020). After coincubation for 48 h, the cells were fixed and lysed by 0.1% Triton-X. Cells were detected using fluorescence microscopy after incubation with phalloidin and DAPI in the dark.

#### 2.6.5 qRT‒PCR

Total cellular RNA was obtained using TRIzol, and RNA purity was determined using a Nanodrop 2000C. RNA was reverse transcribed, and cDNA amplification was performed using a CFX Connect Real Time PCR Detection System (Bio-Rad, Singapore). Supplementary Table S1 showed the primer sequences. The 2^−ΔΔCt^ method was utilized to analyze gene expression levels.

#### 2.6.6 Western blot

Cells were lysed in RIPA buffer on ice and quantified by BCA method. The protein samples with 5× SDS loading buffer were loaded into the wells of the prepared SDS‒PAGE gel. Electrophoresis was performed at 80 V (for the concentrated gel) and 120 V (for the separated gel) until bromophenol blue reached the bottom of the gel. Proteins were transferred to a PVDF membrane, and then the membranes were blocked and incubated with primary and secondary antibodies. The ECL method was used for protein detection. ImageJ software was used to quantify the grayscale values.

### 2.7 *In vivo* animal studies

#### 2.7.1 Animal surgical procedure and administration of the drug delivery system

All animals in this experiment were purchased from Liaoning Changsheng Experimental Animal Company, and all animal work was approved by the Institutional Animal Care and Use Committee of Jilin University School of Pharmaceutical Science (No. 20220034). Twenty-four 12-week-old SD rats (female, 250–280 g) were randomly divided into the sham-operated group (*n* = 3) and the OVX group (*n* = 21). All OVX rats underwent bilateral ovariectomy after anesthesia (intraperitoneal injection of pentobarbital sodium, 45 mg/kg), while a comparable mass of adipose tissue surrounding the ovary was cut off in the sham-operated group. Three months after OVX surgery, three rats in each group were selected, and the distal femurs were harvested for micro-CT examination. The remaining rats were assigned to 3 groups: (1) Con group: OVX + nondrug-loaded scaffold group (*n* = 6); (2) PTH group: OVX + PTH_1-34_ group (*n* = 6); and (3) PTH + SV group: OVX + PTH_1-34_ + SV group (*n* = 6). By a dental trephine, an 8 mm full-thickness circular defect was created on the midline of the rat’s parietal bone under anesthesia. After the delivery system was placed in the bony defect, rats were given penicillin postoperatively for 3 days. Rats were sacrificed by cardiac perfusion under anesthesia at 4 or 8 weeks.

#### 2.7.2 X-ray and micro-CT

To assess the extent of bone repair, X-ray (Faxitron MultiFocus, United States) and micro-CT (Scanco, Switzerland) imaging was performed. The specimens obtained were placed in 4% paraformaldehyde before X-ray and micro-CT analysis. Each planar radiography was scored according to a previous description (Spicer et al., 2012). Using micro-CT at a resolution of 34 μm, regions of interest (ROIs) were selected, and bone volume fraction (BV/TV), bone volume (BV), bone surface area (BS), trabecular number (Tb. N), trabecular thickness (Tb. Th) and trabecular separation (Tb. Sp) were determined.

#### 2.7.3 Histological observation

Rat skull specimens were decalcified and sectioned for HE, Masson’s Trichrome, and immunohistochemical (IHC) staining. Primary antibodies against ALP (1:200, 13365-1-AP, ProteinTech, Wuhan, China), BMP2 (1:200, 18933-1-AP, ProteinTech, Wuhan, China), COL1 (1:800, GB11022-3, Servicebio, Wuhan, China), OCN (1:200, 23418-1-AP, ProteinTech, Wuhan, China), and HRP-labeled secondary antibody (1:200, 5220-0336, SeraCare, Beijing, China) were used. Viscera were harvested to observe structural changes by HE staining. The sections were observed using a photomicroscope (OLYMPUS, BX53).

### 2.8 Statistical analysis

All data are expressed as the mean ± standard. A minimum of 3 parallel samples were tested at each time point. Statistical analysis was performed using GraphPad Prism 8.0 software, utilizing Student’s t-test or analysis of variance (ANOVA) as appropriate. For data with unequal variances or nonnormal distributions, Welch’s *t*-test or the Kruskal‒Wallis nonparametric test was employed. Statistical significance was assigned as * (*p* < 0.05), ** (*p* < 0.01) or *** (*p* < 0.001), respectively.

## 3 Results

### 3.1 Physical characteristics of dual-drug delivery systems

**FIGURE 2:**
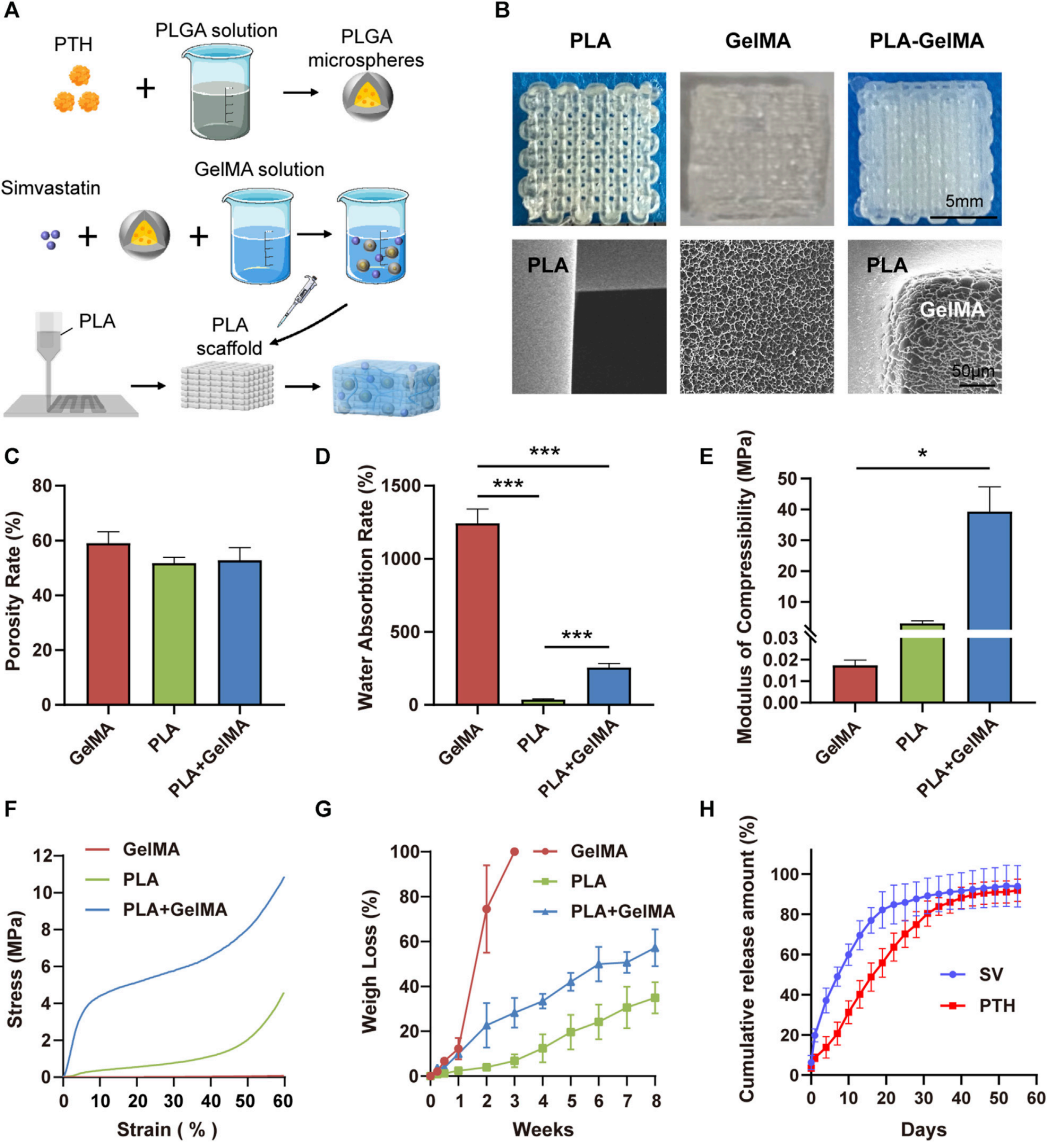
Synthesis and characterization of dual drug delivery systems. **(A)** Schematic diagram of the preparation process of the dual-drug delivery system. **(B)** Macroscopic digital photographs and microscopic morphology of the PLA, GelMA, and PLA-GelMA delivery systems under scanning electron microscopy. The **(C)** porosity rate, **(D)** water absorption rate, **(E)** compression modulus, **(F)** stress‒strain curve, **(G)** degradation weight loss, and **(H)** PTH_1-34_/SV cumulative release curves are shown. * and *** representing *p* < 0.05 and *p* < 0.001 respectively.

We followed the procedure illustrated in Figure 2A to prepare the delivery systems. The photo of PLGA microspheres is shown in Supplementary Figure S1, and the encapsulation efficiency is 72.07% ± 1.89%. Different printing line distances on pore size and fiber size of PLA scaffolds were investigated, as shown in Supplementary Figure S2, and a printing line distance of 1.0 mm was finally selected for subsequent experiments. The gelation process of GelMA is shown in Supplementary Figure S3. The macroscopic and microscopic images of the PLA scaffold, GelMA delivery system, and PLA-GelMA system are shown in Figure 2B. All samples maintained their preset sizes. SEM revealed that the pore size in the 3D-printed PLA scaffold was approximately 500 μm, which was similar to that of natural cancellous bone (300–600 μm) (Anita Lett et al., 2021). The GelMA delivery system demonstrated microporous pores. For the PLA—GelMA composite delivery system, the microporous pores of the GelMA delivery system filled the macroporous pores of the PLA scaffold, which resulted in a close embedding relationship.

In our experiments, the porosity rates of the PLA scaffolds, the GelMA delivery system, and the PLA-GelMA system were 51.74% ± 1.74%, 59.05% ± 3.41%, and 52.82% ± 3.77%, respectively, and no notable disparities were identified among the groups (Figure 2C). The average water absorption rate of the GelMA delivery system was 1242.18% ± 85.65%, while that of PLA was 36.27% ± 4.77%. When the hydrogel was combined with the PLA scaffold, the water absorption rate decreased to 255.88% ± 24.67% (Figure 2D). The loading‒displacement curve and the stress‒strain curve showed that the stress of GelMA was always maintained at a low level, whereas the stress of PLA and PLA-GelMA increased with compression (Figure 2F;Supplementary Figure S4). The compressive modulus data of the GelMA hydrogel, the PLA scaffold, and the PLA-GelMA composite system were 17.377 ± 1.953 kPa, 3.032 ± 0.751 MPa, and 39.318 ± 6.549 MPa, respectively (Figure 2E). This result showed that the incorporation of PLA enhanced the ability of the delivery system to withstand mechanical.

We investigated the degradation characteristics of the three systems in PBS (Figure 2G). Our results showed that all three systems exhibited continuous degradation over time, with the GelMA system showing the fastest degradation rate and reaching complete degradation within 3 weeks. The PLA scaffold had a slower degradation rate and reached a weight loss rate of 34.94% ± 6.04% over 8 weeks. The degradation rate was found to be slow in the initial 3 weeks, but increased in the later stages. The PLA-GelMA system degraded quickly in the first 2 weeks, which was followed by a slower degradation rate in the remaining weeks. In addition, the pH values of all three delivery systems were maintained at physiological levels between 6.8 and 7.3 during degradation (Supplementary Figure S5).

### 3.2 *In vitro* release characteristics

100 ng/mL PTH and 0.2 μg/mL SV were selected for our experiments as a result of previous tests (Supplementary Figure S6). The cumulative release amount of SV and PTH_1-34_ in the composite system is displayed in Figure 2H. The initial release rate of SV was 6.29% ± 2.88%, followed by a gradual decline until day 19. The cumulative release of SV barely increased after 19 days, which was most likely due to complete degradation of the hydrogel. On the contrary, the initial release of PTH_1-34_ was not obvious (3.46% ± 0.08%), which may be because PTH_1-34_ was pre-encapsulated in PLGA microspheres. In addition, the cumulative release of PTH_1-34_ achieved a zenith around day 40. The release results showed that the composite system could allow a prolonged release of the drugs and could successfully deliver both PTH_1-34_ and SV *in vitro*, which was favorable for bone healing in a pathological environment.

### 3.3 Synergistic osteogenic effects of the dual-drug composite delivery system *in vitro*

**FIGURE 3:**
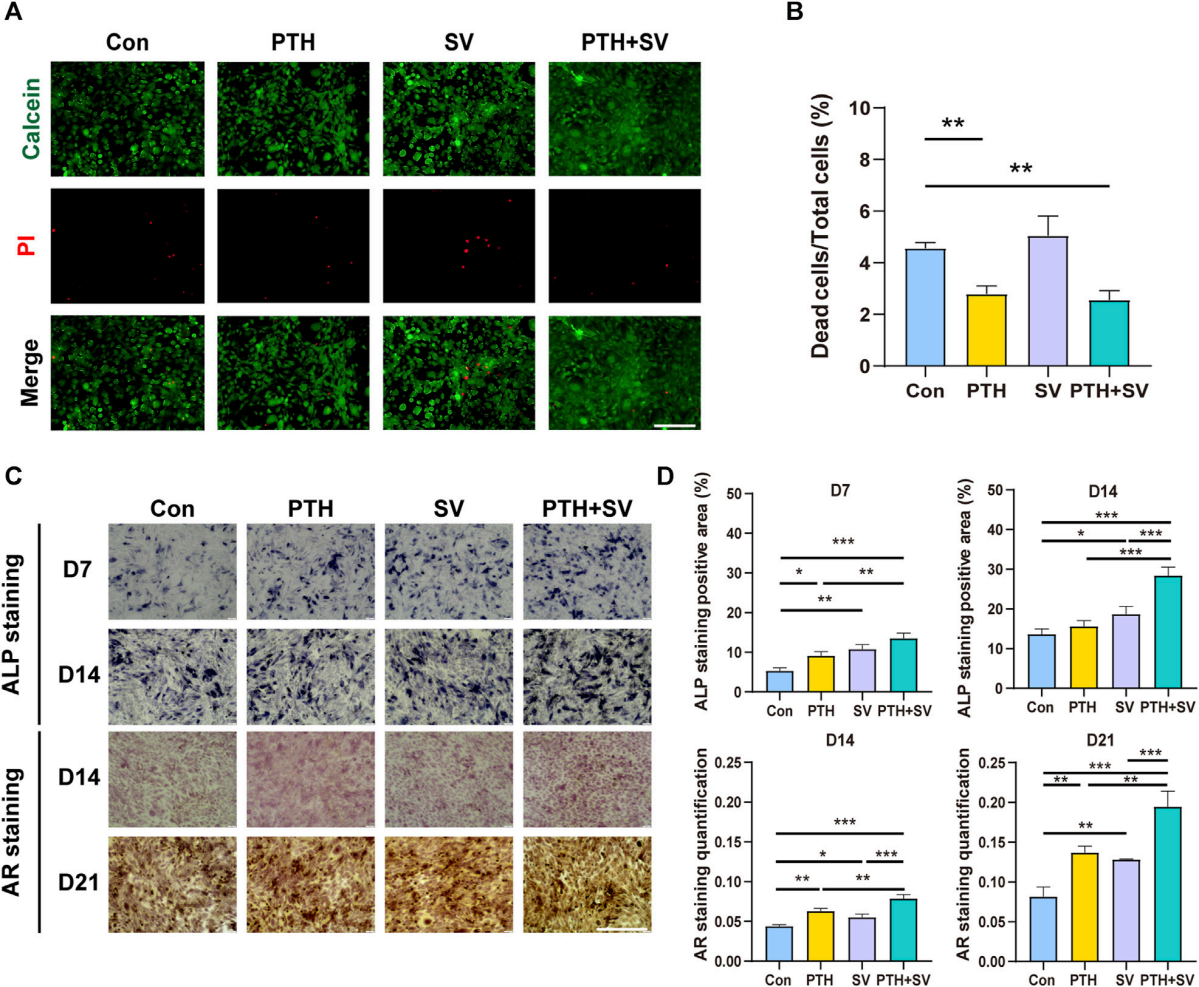
Effect of dual administration of PTH_1-34_ and SV on the survival and differentiation of MC3T3-E1 cells. **(A)** Live/dead staining images. The scale bar is 200 μm. **(B)** Quantitative analysis of the cell death ratio. **(C)** ALP and AR staining images. The scale bar is 500 μm. **(D)** Quantitative analysis of ALP activity and calcium nodules. *, **, and *** representing *p* < 0.05, *p* < 0.01, and *p* < 0.001, respectively.

To investigate the effectiveness of composite delivery systems containing PTH_1-34_ and/or SV, studies on the survival and differentiation status of MC3T3-E1 osteoblasts were conducted. Live/dead staining data indicated that the SV group and the control group had similar levels of dead cells (red fluorescence), as shown in Figures 3A, B. However, the PTH and PTH + SV groups showed a lower rate of cell death. Overall, all the mortality rates were far less than 30%, which indicated no cytotoxicity according to GB/T 16886.5-2017. To further investigate the combined effect of PTH_1-34_ and SV on osteogenic differentiation, we performed ALP staining and mineralized nodule staining (Figures 3C, D). Seven and 14 days of osteogenic induction in both the PTH group and the SV group resulted in darker ALP staining, while the darkest staining was found in the PTH + SV group. AR staining revealed enhanced mineralization in all three experimental groups, and the PTH + SV group showed the most obvious mineralization.

**FIGURE 4:**
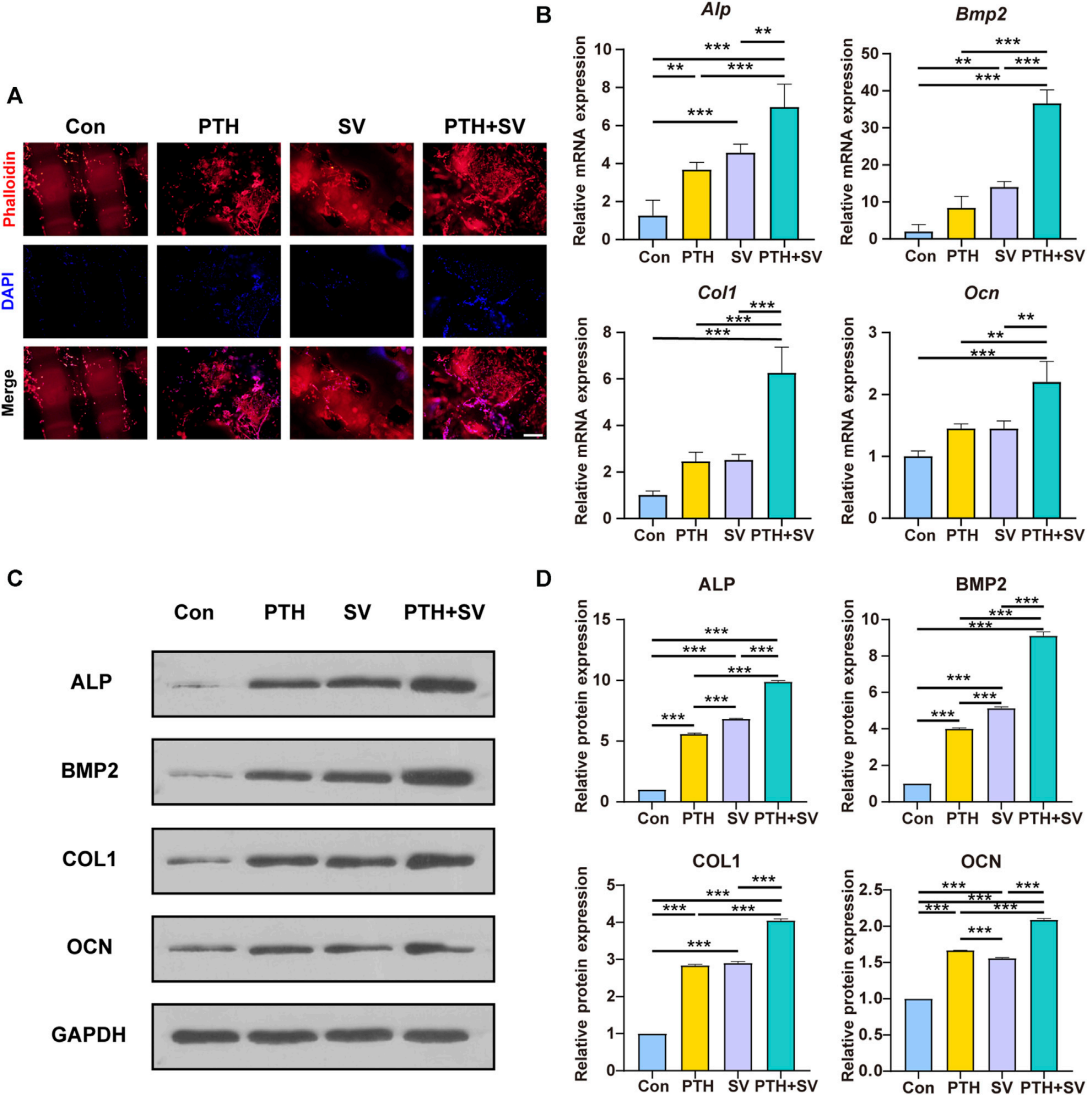
Effect of different delivery systems on adhesion and osteogenic marker expression in MC3T3-E1 cells. **(A)** Fluorescence images of MC3T3-E1 cells cultured on different systems (red: F-actin; blue: nucleus). The scale bar indicates 500 μm. **(B)** Osteogenic gene expression levels in MC3T3-E1 cells. **(C)** Osteogenic protein expression levels and **(D)** quantitative analysis. ** and *** representing *p* < 0.01 and *p* < 0.001 respectively.

The adhesive behaviors of the MC3T3-E1 cells cocultured on different composite systems were observed by examining fibrillar actin and nuclei. The results of rhodamine-phalloidin and DAPI staining showed that MC3T3-E1 cells were able to grow on all composite delivery systems (Figure 4A). The cells had a shuttle shape, with cell protrusions interconnected with each other in a reticular pattern. The counts of red-stained actin and blue-stained nuclei were observed to be elevated in the PTH-containing groups. The *qRT-PCR* analysis indicated that *Alp*, *Bmp2*, *Col1*, and *Ocn* genes were upregulated in both the PTH group and the SV group, while these genes were expressed at the highest levels in the PTH + SV group (Figure 4B). Consistent with the trend of the *PCR* assay results, the expression of related osteogenic proteins was also significantly upregulated after PTH and SV co-loading (Figures 4C, D). The above results indicated that the dual-drug strategy of PTH_1-34_ and SV from the composite delivery system could exert osteogenic effects *in vitro* and that the comedication therapeutic effect was better than that of single-drug treatment.

### 3.4 Dual-drug composite delivery system promotes repair of cranial defects *in situ* in osteoporotic rats

**FIGURE 5:**
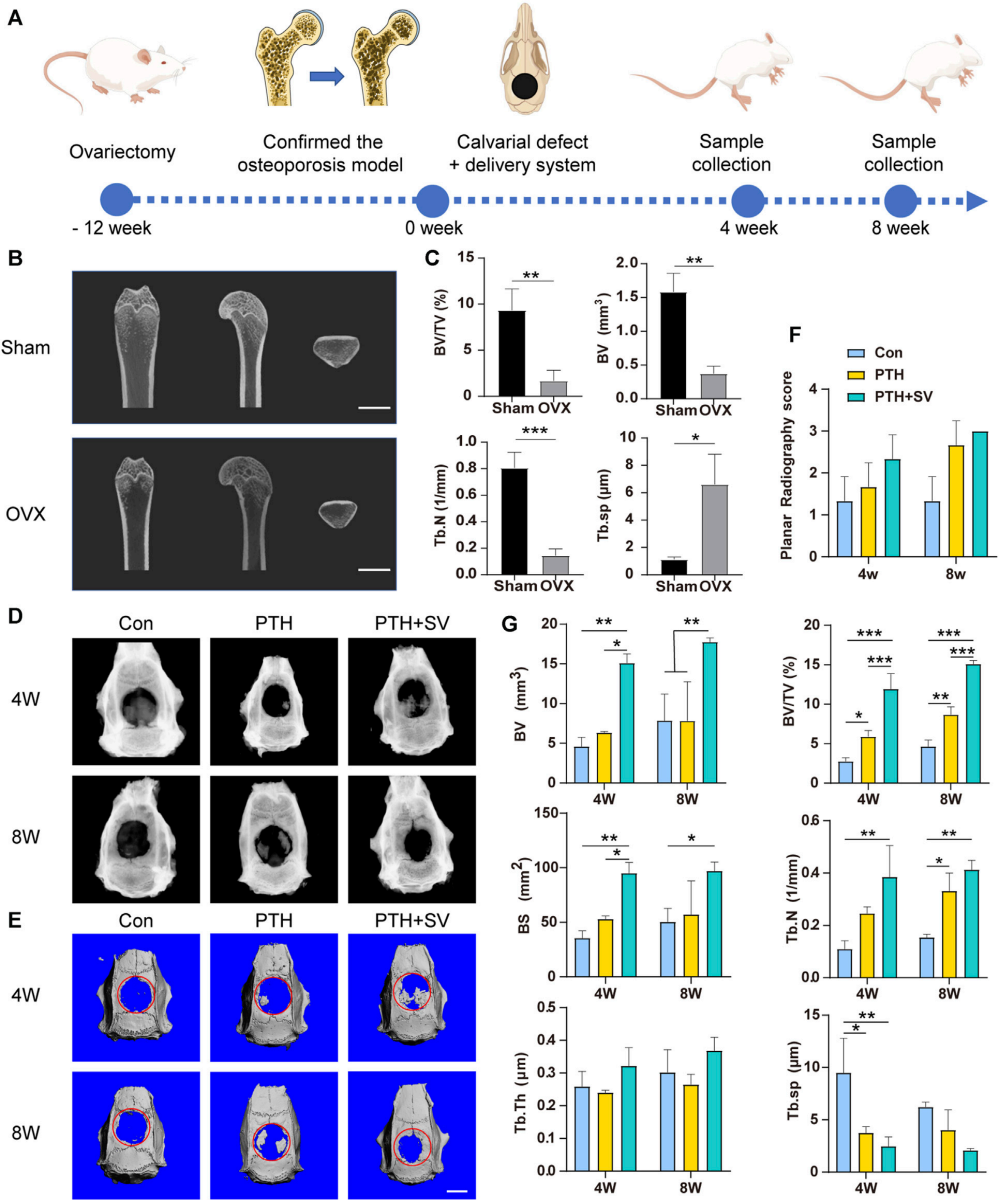
The osteogenic capacity of the dual-drug delivery systems *in vivo*. **(A)** Illustration of the schedule of the animal experimental procedure. **(B)** Micro-CT snapshots of the distal femur of sham and OVX rats and **(C)** analysis results. **(D)** Radiographs and **(E)** micro-CT 3D-reconstructed images of the skull defect after 4 and 8 weeks. **(F)** Planar radiographic score of the skull and **(G)** quantitative micro-CT analysis. The scale bars are 5 mm *, **, and *** representing *p* < 0.05, *p* < 0.01, and *p* < 0.001, respectively.

Osteoporotic bone defects are usually difficult to repair, therefore, we established an osteoporotic model to evaluate the ability of the dual-drug composite delivery system to repair osteoporotic bone defects. The hemolysis rate was below 2% (Supplementary Figure S7), which is considered biosafety for implantation *in vivo* according to ASTM standards (Anita Lett et al., 2021). The entire animal experimental schedule is summarized in Figure 5A, and the surgical procedure is shown in Supplementary Figures S8, S11. In OVX rats, the volume and density of bone trabeculae in the distal femur were decreased, and the bone marrow cavity was expanded (Figure 5B). Quantitative analysis revealed that BV, BV/TV, and Tb. N values demonstrated reductions compared to the sham group, whereas Tb. Sp displayed an elevation in the epiphysis (Figure 5C). Furthermore, the body weight was increased (Supplementary Figure S9), and the uterus was atrophied (Supplementary Figure S10) in OVX rats. The above results demonstrated the successful establishment of the osteoporosis model.

4 and 8 weeks after the delivery system was implanted, all the scaffolds remained at the defect without detachment. X-ray images revealed varying degrees of novel bone in the rat skull in both the PTH group and the PTH + SV group, whereas minimal novel bone was found in the control group (Figure 5D). Both the PTH groups exhibited improved planar radiography scores after 4 and 8 weeks, although the difference was not statistically significant (Figure 5F). As shown in the micro-CT 3D-reconstructed images, almost no increase was observed in the defects in the control group, while island-like and irregular new bone were seen in the other two groups (Figure 5E). BV/TV, BV, BS, and Tb. N values increased in the PTH + SV group, and Tb. Sp values decreased at 4 and 8 weeks. However, no differences were observed in Tb. Th among the three groups (Figure 5G).

**FIGURE 6:**
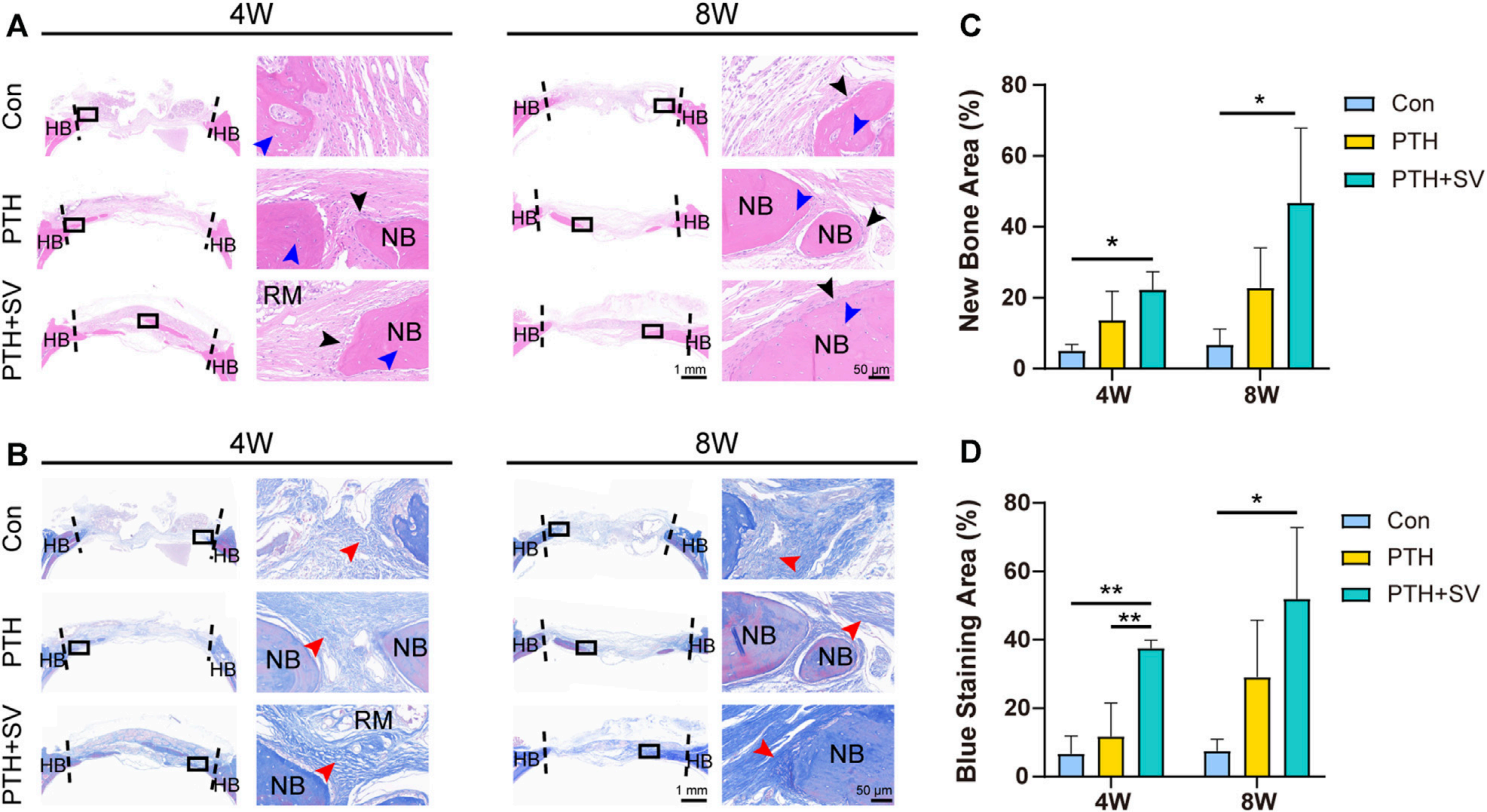
Histological photographs of the skull defect at 4 and 8 weeks. **(A)** HE staining. The black arrow indicates osteoblasts, and the blue arrow indicates osteocytes. **(B)** Masson’s trichrome staining. The red arrow indicates collagen fibers. **(C)** New bone area fraction and **(D)** blue staining area fraction. The scale bars at low magnification (×1) and high magnification (×40) are 1 mm and 50 μm, respectively. HB: host bone, NB: new bone, RM: remaining materials. * and ** representing *p* < 0.05 and *p* < 0.01 respectively.

**FIGURE 7:**
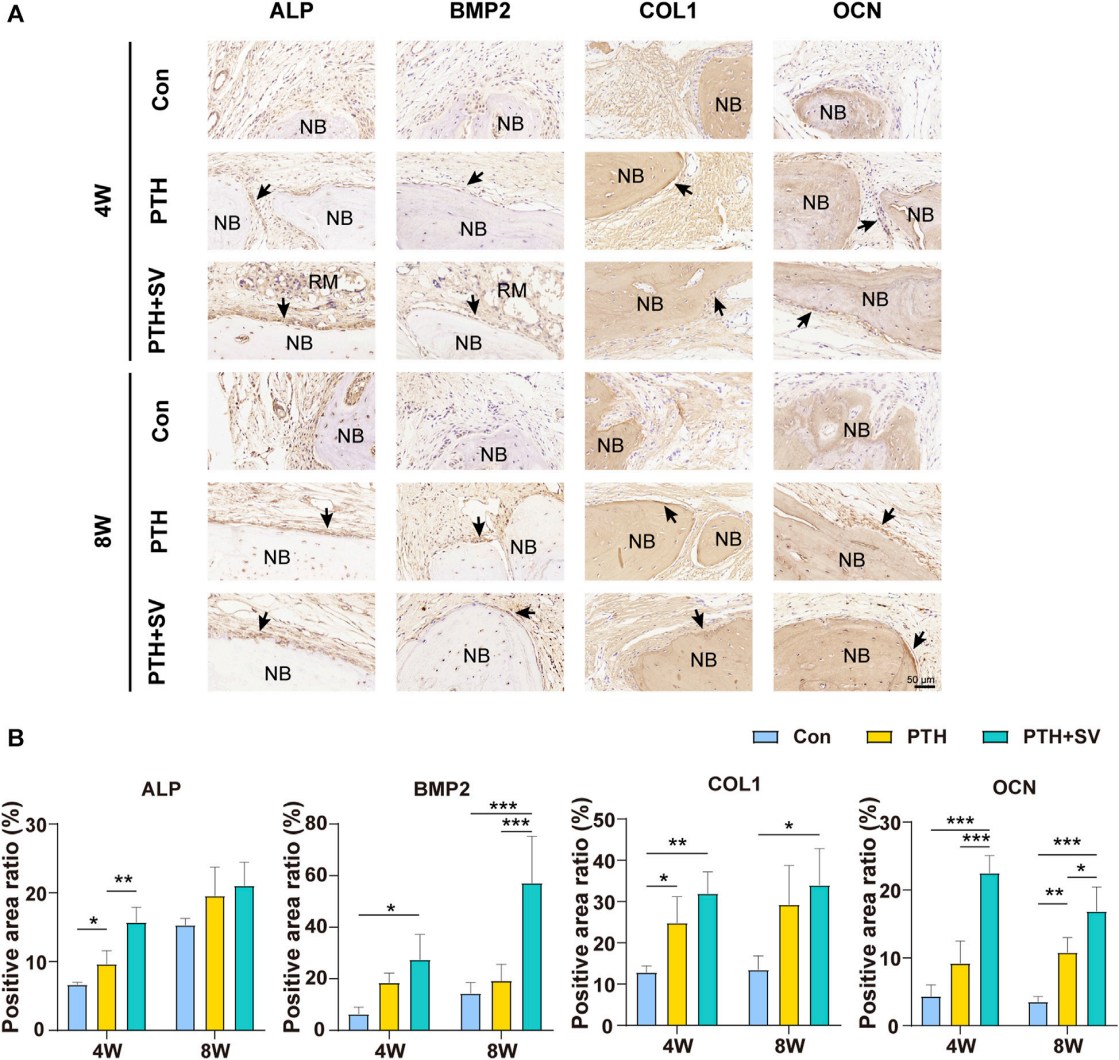
IHC staining at 4 and 8 weeks. **(A)** Expression of ALP, BMP2, COL1, and OCN proteins in rat cranial bone. The scale bar is 50 μm. **(B)** Quantitative analysis of IHC staining. NB: new bone, RM: remaining materials. *, **, and *** representing *p* < 0.05, *p* < 0.01, and *p* < 0.001, respectively.

In the histological observation by HE staining (Figures 6A, C), the defect was replenished with a fibrous layer of sparse reticular fibrous tissue in the control group. Conversely, several islands of bone-like structures were visible in the reticular fibrous tissue in the PTH group, and osteoblasts and osteocytes could be seen. More novel bone was observed to connect to the host bone or through the material and connective tissue in the PTH + SV group, which resulted in a significantly larger area of new bone and more osteoblasts. By 8 weeks, all groups showed similar growth trends but exhibited greater bone mass and less residual material compared with groups at 4 weeks. Masson’s trichrome staining (Figures 6B, D) demonstrated that the defects consisted of sparse and disordered collagen in the control group, whereas the collagen was denser and more orderly in the PTH and PTH + SV groups. IHC staining (Figure 7) showed that a greater extent of positive staining for ALP, BMP2, COL1, and OCN in the PTH group, and this increase in positive staining was particularly noticeable in the PTH + SV group.

Finally, we conducted a toxicological evaluation of the dual-drug composite delivery system (Supplementary Figure S12). The sections of the viscera from rats who received the delivery system revealed no significant abnormalities, which indicated that the dual-drug composite delivery system does not induce any toxic reactions.

## 4 Discussion

Osteoporosis is a prevalent metabolic bone disorders that affects a significant number of individuals worldwide, resulting in pain, fractures, and substantial annual costs. Worse still, impaired bone regeneration also hinders the repair process after trauma or infection. Therefore, a functional composite system carrying bioactive factors or drugs would provide a good repair strategy for difficult-to-heal bone defects.

Ideal osteogenic biomaterials must possess several characteristics, such as biocompatibility, suitable shape and porosity, matched mechanical force, osteoconductivity, osteoinductivity, and degradability (Wang S. et al., 2021; Sang et al., 2022; Xue et al., 2022). Additionally, a delivery system that serves as a local platform to sustainably release active substances should be considered. GelMA is an ideal platform for controlled drug release and cell adhesion (Sun et al., 2018; Collins et al., 2021; Kimicata et al., 2021). The hydrogels establish a biomimetic microenvironment for bone tissue engineering (Meng et al., 2022; Xue et al., 2022), but their biomechanical properties are slightly inadequate compared with those of hard bone tissue. To reinforce the mechanical strength of GelMA, secondary structural scaffolds are typically incorporated into the hydrogel matrix (Boere et al., 2014). Here, we employed PLA as the secondary scaffold due to its strength properties and 3D printable properties, and the combined design of polymer and hydrogel provides a biomimetic organoid for bone regeneration (Xue et al., 2022). Our composite drug delivery system achieved satisfactory results in terms of physical properties. First, personalized irregular and regular tissue defects can be perfectly replicated using a 3D-printed scaffold and a light-curing hydrogel, both of which can combine efficiently. Second, the physical parameters of the delivery system, such as porosity and degradability, are comparable to those of natural bone, and similar physical properties will be conducive to subsequent cell behavior and nutrient exchange in the environment. The porosity of the delivery systems ranged from 51.74% to 59.05%, which is consistent with the porosity of cancellous bone reported in the literature (Collins et al., 2021). We also found a high absorption rate in GelMA and a low absorption rate in the PLA scaffold, while a moderate water absorption rate was found in the PLA- GelMA system. We speculated that this result was related to the incorporation of the internal hydrophobic PLA scaffold, which acted as a “cage” to confine the swelling of the GelMA hydrogel. Therefore, the composite system could absorb exudate and nutrients while maintaining its macroscopic morphology and porosity. The appropriate degradation time of scaffolds contributes to the stability of the bone healing space in the early stage as well as the replacement of bone tissue in the later stage. We observed accelerated degradation during the later stages of GelMA and PLA, which could be because the eroded material created larger pores and increased the contact area over time. GelMA degrades rapidly within 1 month (Wang F. et al., 2021), whereas PLA degrades in 70 days- 5.5 years (Saini et al., 2016; Gasparotto et al., 2022). Therefore, the PLA-GelMA system demonstrated a marked initial degradation but subsequently decelerated in the later stage of degradation due to GelMA depletion. The advantage of this is that even if GelMA degrades rapidly in the first stage, the presence of PLA in the composite system can still provide ongoing support for new bone growth. Third, the mechanical characters of hydrogels (∼5–110 kPa) are generally inferior to those exhibited by natural bone (∼0.2–80 MPa) (Zhao et al., 2016; Zhou et al., 2021), thereby augmenting a risk of material collapse during subsequent tissue regeneration. A 3D-printed PLA scaffold structure can improve the support of biological cue-rich hydrogels (Han et al., 2020; Wang et al., 2022). That is, the PLA-GelMA composite delivery system in this study integrated the characteristics of PLA and GelMA and met the physicochemical requirements of bone scaffolds. In addition, drugs encapsulated in the GelMA hydrogel could achieve sustained release, which can provide continuous stimulation for subsequent treatment.

During bone formation, osteoblasts undergo four stages: proliferation, extracellular matrix maturation, mineralization, and osteoblast apoptosis. The increased number of osteoblasts and their degree of differentiation contribute to the final structure and quality of bone tissue. PTH_1-34_ has been approved by the FDA as a clinical drug for the treatment of osteoporosis. PTH_1-34_ interacts with PTH1R on the surface of osteoblasts and osteocytes and contributes to expanding the number of osteoblasts by reducing apoptosis and exerting an anabolic effect on bone (Silva and Bilezikian, 2015; Chen et al., 2021). Previous studies have used PTH in combination with bisphosphonates to achieve better osteogenesis and reduce the amount of PTH in the treatment of osteoporosis (Zhang and Song, 2020; Zhou et al., 2022). However, one risk of bisphosphonates is medication-related osteonecrosis of the jaw (Pires et al., 2005). Statins have a similar effect to bisphosphonates in inhibiting the mevalonate pathway, but carry no risk of osteonecrosis and promote osteogenesis through multiple signaling pathways (Xia et al., 2018; Jin et al., 2021; Guo et al., 2022). Previous studies reported that the combined use of two drugs resulted in a greater osteogenic effect, but the drugs in these studies were administered systemically (Jeon and Puleo, 2008; Tao et al., 2015; Tao et al., 2016). In this investigation, we examined the combined influences of PTH and SV on osteoblasts and osteoporotic bone defects *in situ* via the PLA-GelMA composite delivery system. According to the cell viability assay and differentiation experiments, 100 ng/mL PTH_1-34_ and 0.2 μg/mL SV were demonstrated to be safe for osteoblasts and exerted superior osteogenic effects, as such, these concentrations were used in subsequent experiments. We further observed the biological synergistic effect of PTH_1-34_ and SV on osteoblasts using the composite delivery system. Our results suggested that the cell death rates in the PTH and PTH + SV groups were slightly reduced, while osteogenic differentiation was significantly improved in all experimental groups. Bone formation is highly correlated with the number of osteoblasts and their level of differentiation. The number of osteoblasts was expanded by PTH administration, which led to increased ALP expression and mineralization. In addition, SV promoted osteogenic differentiation, which further improved bone formation. For this reason, the PTH + SV group showed the most significant enhancement in ALP activity and calcium nodule staining.

Immunofluorescence staining showed that the cocultured MC3T3-E1 cells were able to adhere to all composite scaffolds, which is beneficial for subsequent bone formation. The PTH group and the PTH + SV group displayed higher cell counts, possibly related to the reduction in apoptosis induced by PTH administration (Silva and Bilezikian, 2015; Chen et al., 2021; Jin et al., 2021). We also conducted a study to examine the expression of osteogenesis-related genes and proteins, and the results were consistent with the ALP and AR staining mentioned above. *Alp* is a representative early osteogenic marker, while *Ocn* serves as a representative late osteogenic marker. *Bmp2* induces bone formation and regeneration during early embryonic development, and *Col1* is the predominant collagenous fiber in the bone matrix (Zhang Y. et al., 2022). According to our experimental data, the combination of PTH_1-34_ and SV upregulated osteogenic genes and proteins, which indicates that they contribute to early and late osteogenesis. Notably, a substantial rise in *Bmp2* gene expression was observed in the SV group, while PTH expression was not significantly different. This phenomenon might be credited to the functional role of SV in eliciting osteogenic differentiation via enhanced BMP2 expression (Mundy et al., 1999; Kodach et al., 2011).

Using *in vivo* experiments, we first established an osteoporosis model in rats by performing bilateral ovariectomy. The decreased volume of femoral trabecular bone, along with the increased weight and atrophic uterus, confirmed the successful establishment of the model. An 8 mm rat cranial defect is defined as a typical critical bone defect (Spicer et al., 2012). Indeed, the control group in this study showed minimal new bone formation. Although the delivery system is theoretically osteoconductive, the impaired osteogenic capacity in osteoporotic conditions still prevents bone formation. When the anti-osteoporotic factors were loaded, a greater increase in new bone production was noted. Histological staining at 4 and 8 weeks revealed that the PTH + SV group exhibited the most extensive new bone area as well as the greatest collagen content. Additionally, this group also showcased the highest extent of immunopositive IHC staining for ALP, BMP2, COL1, and OCN, which concurred with the findings obtained from *in vitro* experimentation. Furthermore, the hemolysis test and HE staining of various organs revealed no obvious hemolysis reaction or tissue toxicity as a result of any of the composite systems, which demonstrates the biosafety of *in vivo* implantation.

## 5 Conclusion

In summary, this study aimed to design a personalized biomimetic local drug delivery system to repair bone defects in osteoporosis. For the first time, this novel approach applied PTH and SV in combination for *in situ* bone defects. The dual-drug delivery system can be summarized by the following characteristics. First, 3D-printed PLA scaffolds and injectable light-curing hydrogels could replicate irregular shapes to better conform to the unique profile of bone defects. Second, the composite delivery system has physical properties that match those of natural bone tissue and successfully achieved dual-controlled release of PTH_1-34_ and SV. Third, the cytological, radiological, and histological analyses of the drug delivery system demonstrated its safety and efficacy. The dual release of the active molecules PTH_1-34_ and SV synergistically enhanced the repair of osteoporotic defects, possibly through an increased cell counts and an enhanced differentiation of osteoblasts.

However, despite our promising results, we also acknowledge that our work has some limitations. The cranial bone defect did not fully recover within the 8-week observation period. Therefore, future investigations should consider additional factors, longer observation times, and additional underlying mechanisms. In addition, the concentrations of PTH_1-34_ and SV were screened separately through *in vitro* experiments, and an appropriate combination of the two drug doses might yield more optimized results. Nevertheless, these limitations do not undermine the potential of the dual-drug delivery system to promote bone regeneration through the release of PTH_1-34_ and SV. Consequently, this localized personalized biomimetic dual-drug composite delivery system presents an encouraging and safe approach for the medical therapeutics of bone defects in individuals with osteoporosis.

## Data Availability

The original contributions presented in the study are included in the article/Supplementary Material, further inquiries can be directed to the corresponding authors.
