# Supplementary material for: A personalized biomimetic dual-drug delivery system via controlled release of PTH1-34 and simvastatin for in situ osteoporotic bone regeneration
Source: Front Bioeng Biotechnol. 2024 Jan 31;12:1355019. doi: 10.3389/fbioe.2024.1355019 (PMC10865375; doi:10.3389/fbioe.2024.1355019)
Supplement: Supplementary file 1 [file DataSheet1.pdf]

Supplementary Material

1 Supplementary Figures

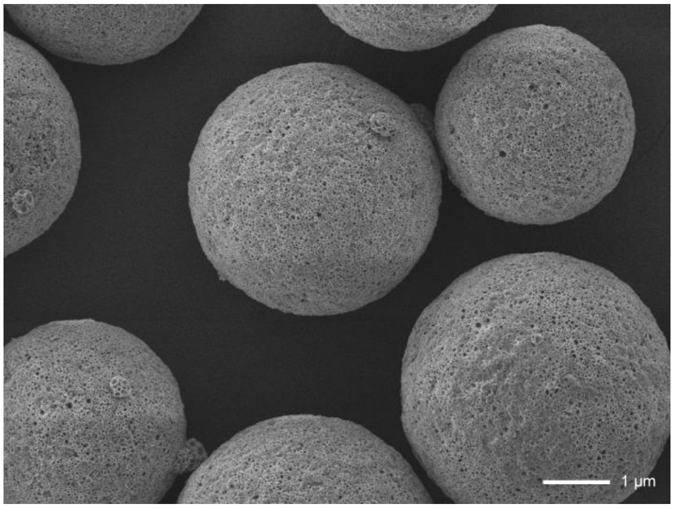

Supplementary Figure 1. SEM photo of PTH microspheres. The average diameter of the microspheres was determined to be 3.6 ± 0.42 μm.

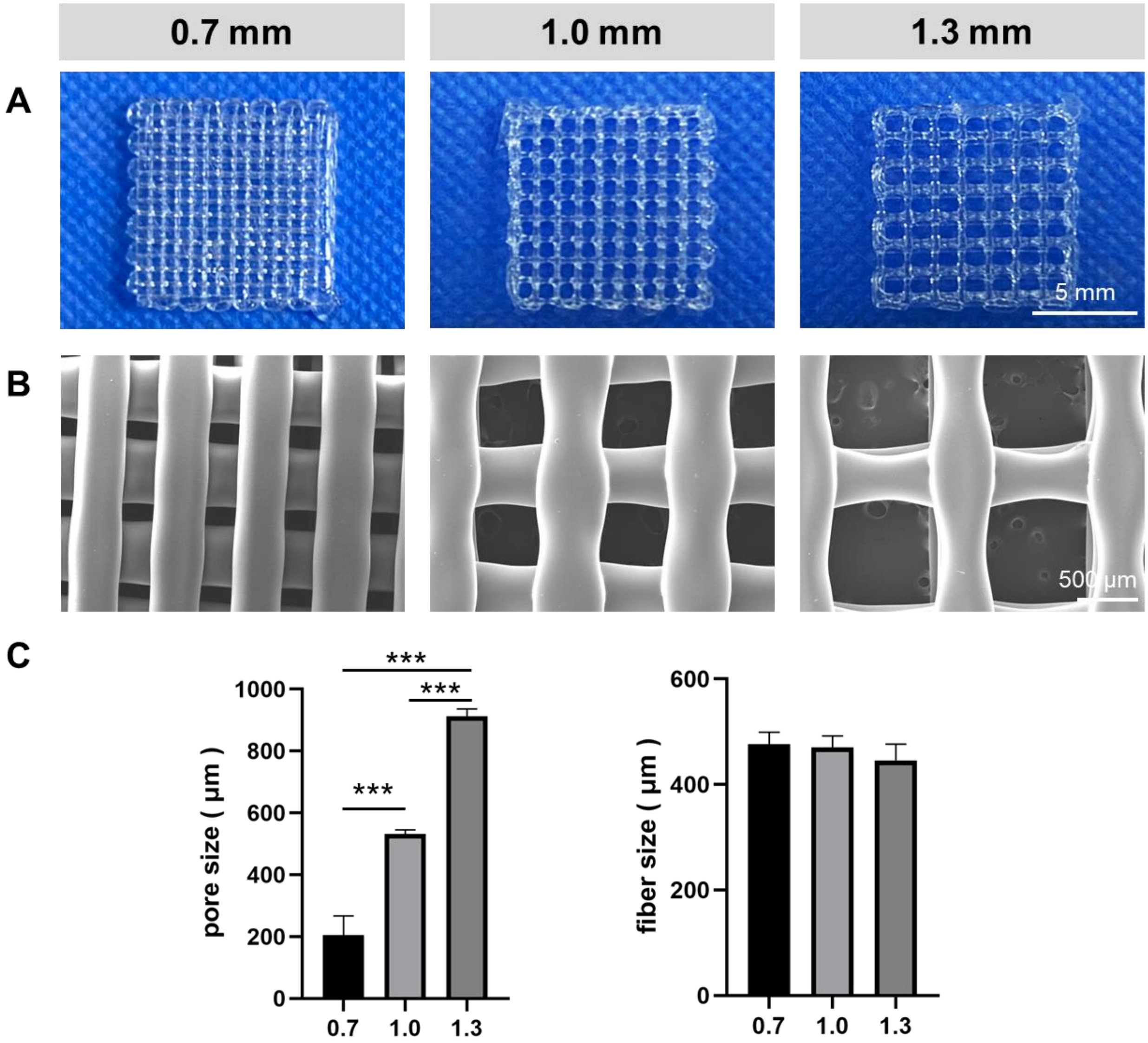

Supplementary Figure 2. Effect of different line distances (0.7 mm, 1.0 mm, 1.3 mm) on the porosity of 3D printed PLA scaffolds. (A) General observation of the printed PLA scaffolds with different line distances. (B) Scanning electron microscopy results of the scaffolds with different print parameters. The printed fibers are all relatively uniform and no broken lines are seen. The printed filament lines are all relatively uniform and no fracture lines are seen. (C) The average pore size of the stents and the average diameter of the printed fibers. Error bars indicate standard deviation (n=6), and *** representing $p < 0.001$.

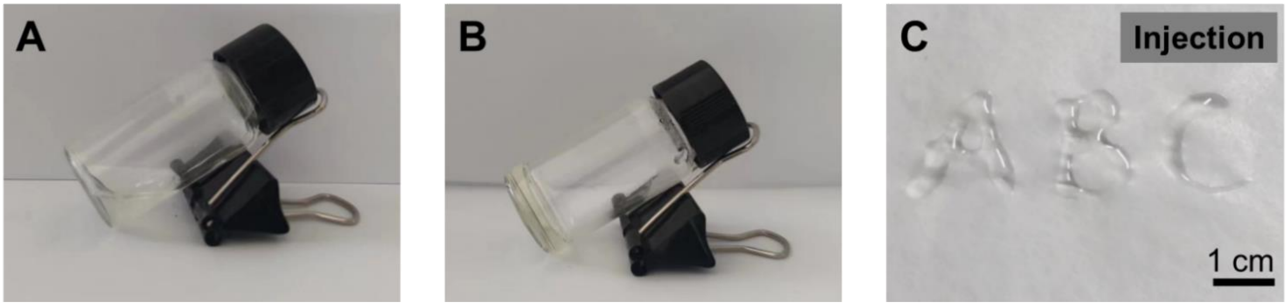

Supplementary Figure 3. Visualization of the sol-gel injection process. The GelMA solution was free-flowing before UV irradiation(A), and turned into a gel after exposed to the UV radiation (B). (C) To assess the injectability of the synthetic hydrogels, the letters "ABC" was written by continuous injection with a syringe.

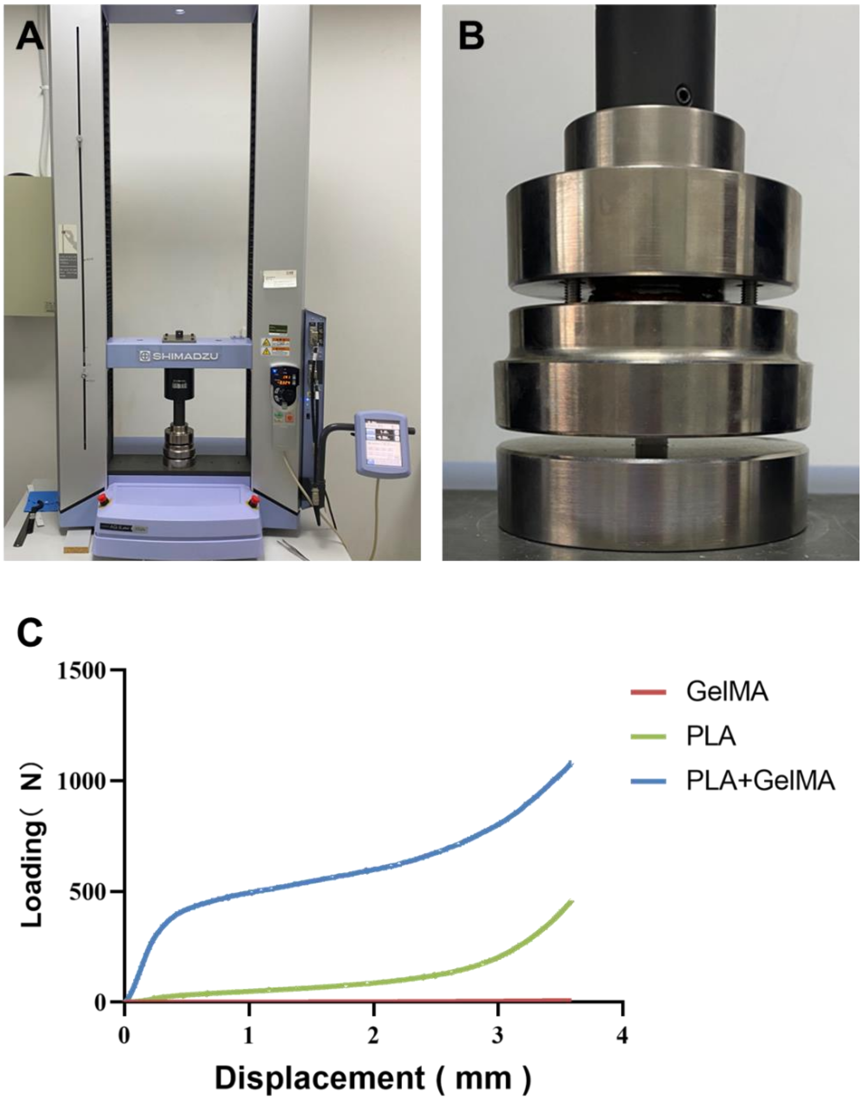

Supplementary Figure 4. Photographs of different samples tested on a universal testing machine (A, B) and displacement load curves (C).

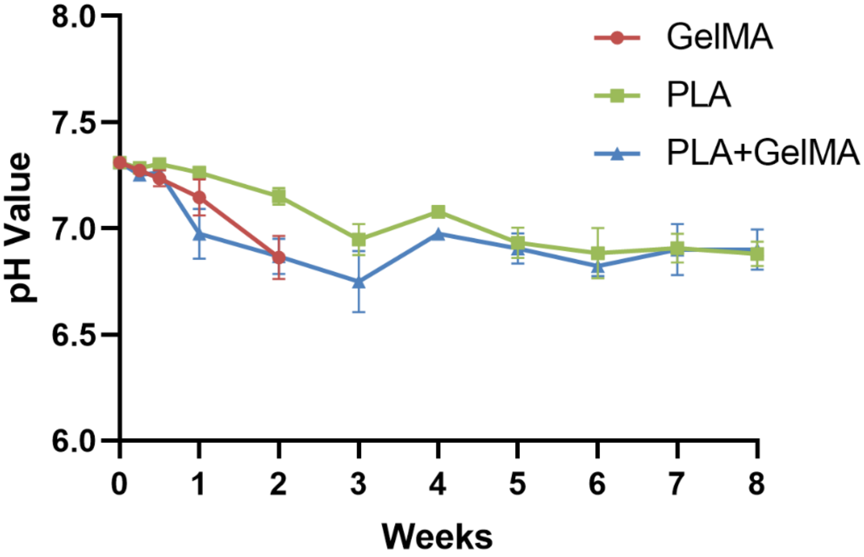

Supplementary Figure 5. The pH value of different samples.

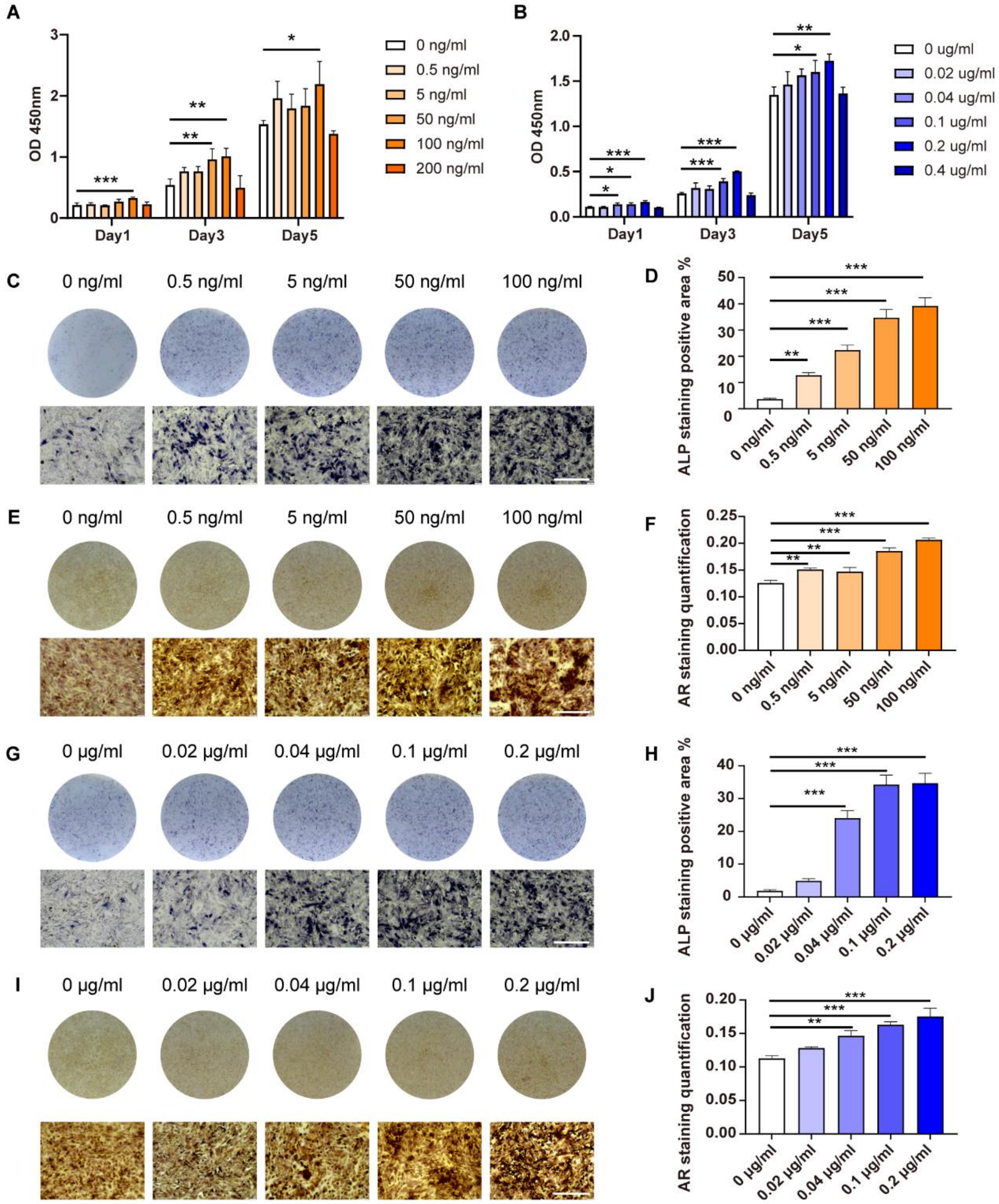

Supplementary Figure 6. Effective concentrations of PTH_{1-34} and SV for osteogenesis. (A) The cell viability of MC3T3-E1 at day 1, day 3, and day 5 under different concentrations of PTH_{1-34}. (B) The cell viability of MC3T3-E1 at day 1, day 3, and day 5 under different concentrations of SV. (C and D) ALP activity of MC3T3-E1 and quantitative analysis under different concentrations of PTH_{1-34}. (E and F) AR activity of MC3T3-E1 and quantitative analysis under different concentrations of PTH_{1-34}. (G and H) ALP activity of MC3T3-E1 and quantitative analysis under different concentrations of SV. (I and J) AR activity of MC3T3-E1 and quantitative analysis under different concentrations of SV. The scale bar indicates 500 μm. *, **, and *** representing $p < 0.05$, $p < 0.01$, and $p < 0.001$, respectively, relative to the 0 ng/ml group.

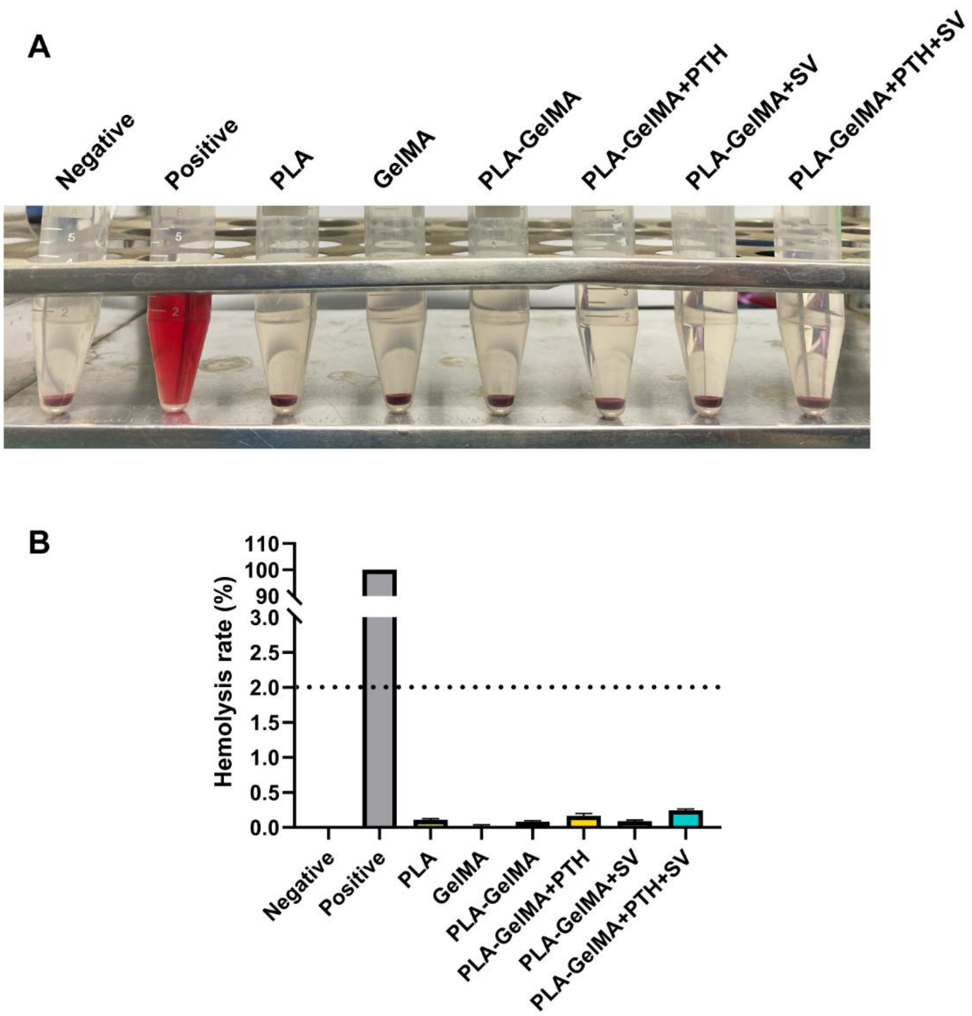

Supplementary Figure 7. The hemolysis and corresponding hemolytic ratio of different samples. From left to right are negative group (PBS), positive group (distilled water), PLA, GelMA (PLGA microsphere-containing), PLA-GelMA, PLA-GelMA+PTH, PLA-GelMA+SV, and PLA-GelMA+PTH+SV, respectively. (A) The photos of the test tube. (B) The hemolysis rates.

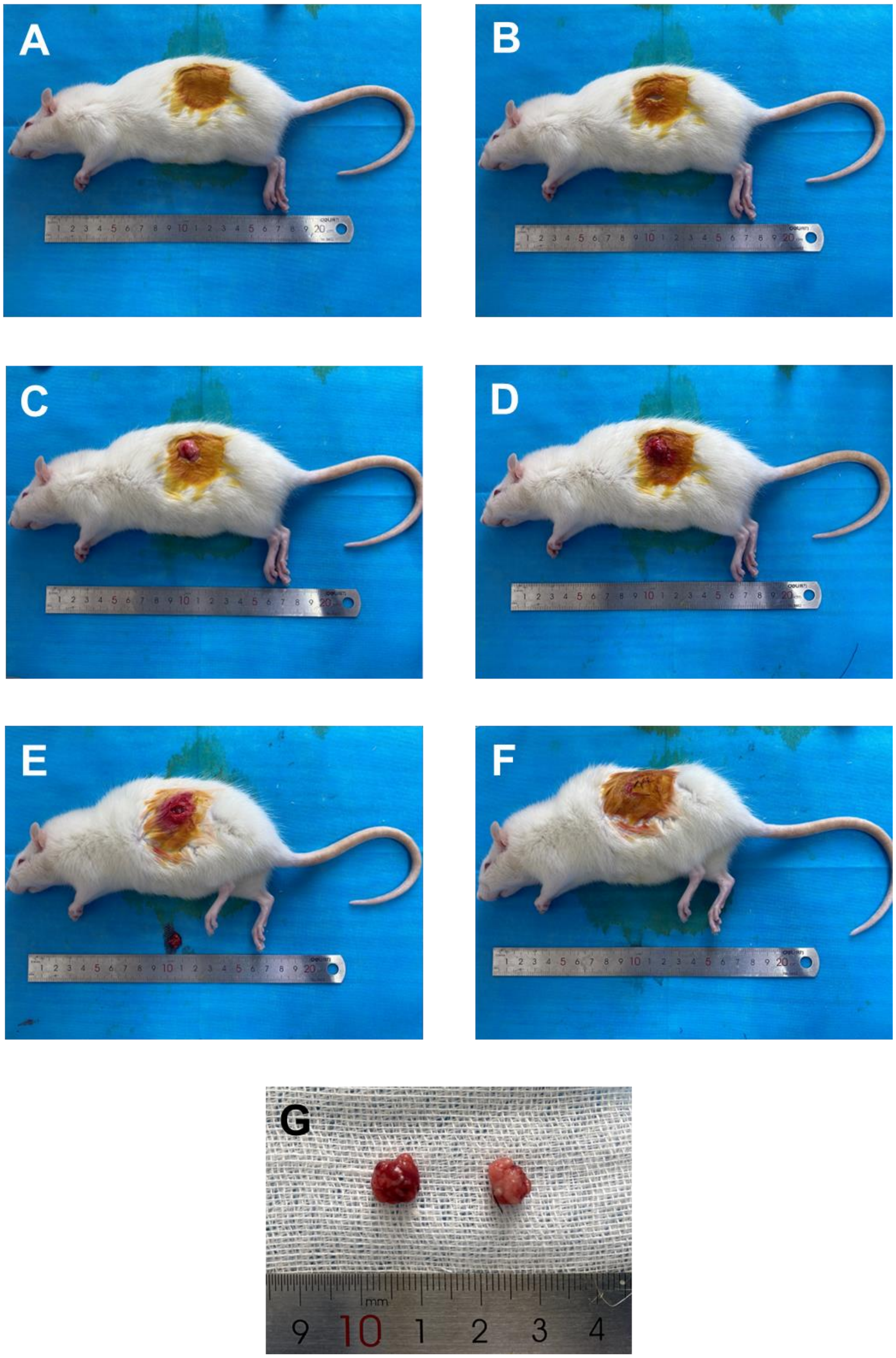

Supplementary Figure 8. OVX surgical procedure. (A) Rat skin preparation and disinfection; (B) Left dorsal incision; (C) Exposure of the ovary and adipose mass; (D) Ligation of the ovary; (E) Removal of the ovary; (F) Layered suture of dorsal skin and muscles; (G) Excised bilateral ovaries.

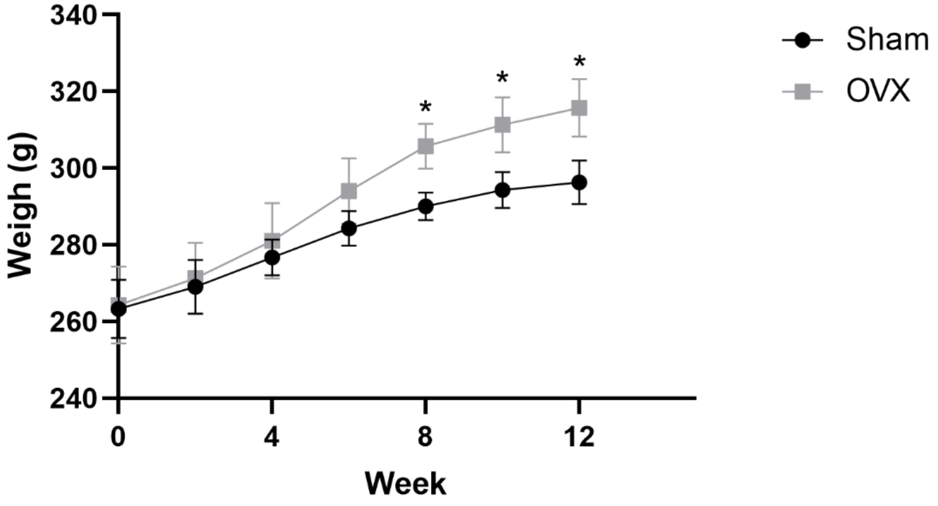

Supplementary Figure 9. The body weight curve of rats. Week 0 represents the day of OVX operation. * representing p<0.05.

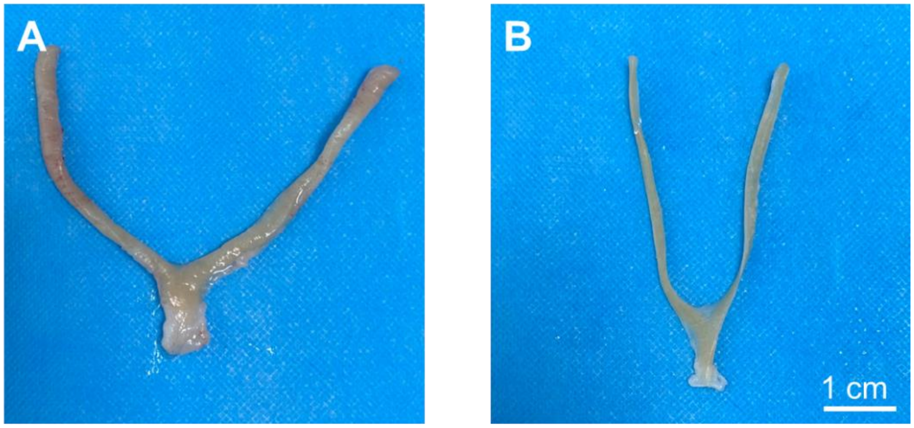

Supplementary Figure 10. Gross photograph of the uterus. (A) Uterus of normal group; (B) Uterus at 3 months after bilateral ovarian removal. The scale bar indicates 1 cm.

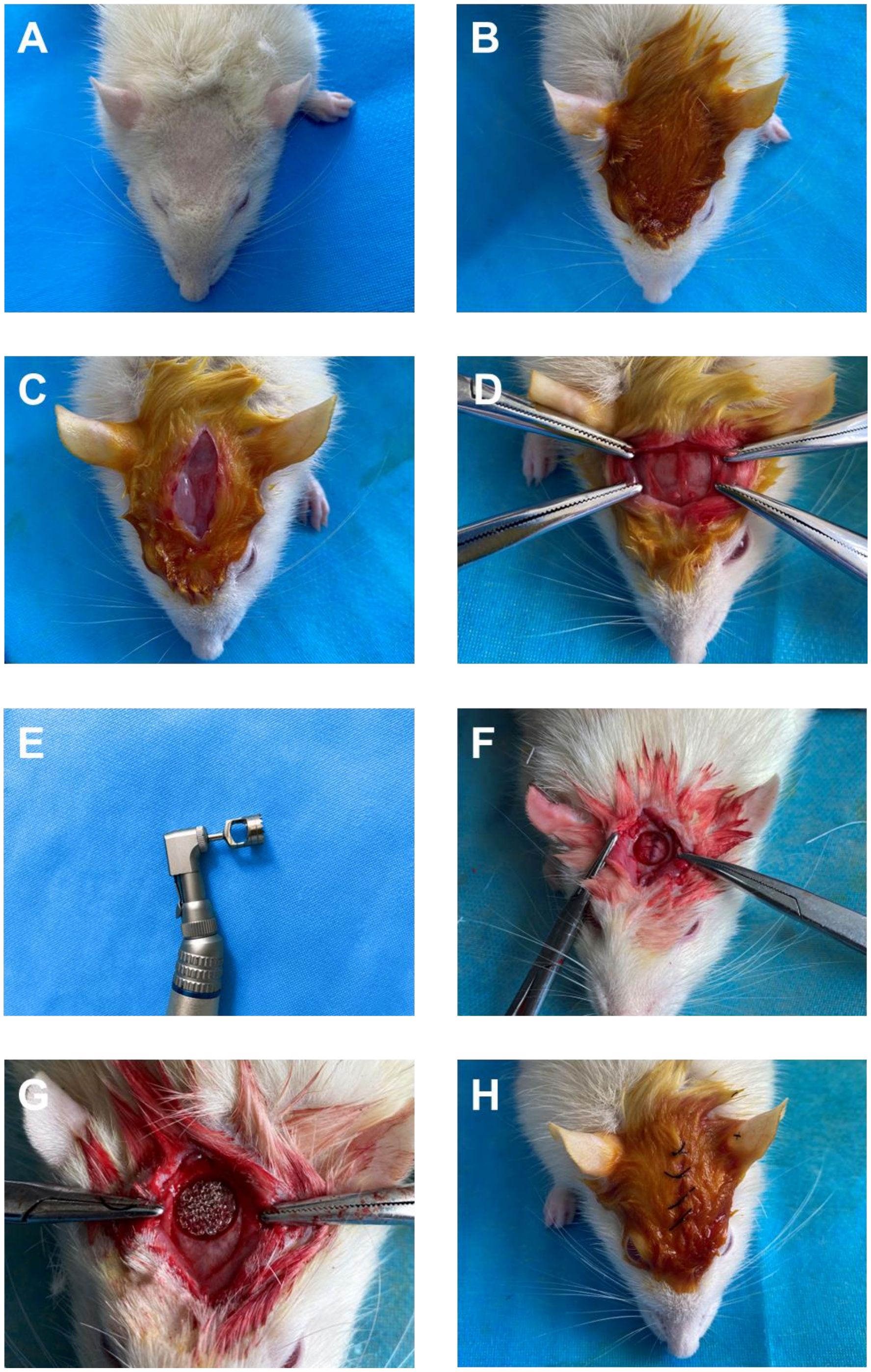

Supplementary Figure 11. Cranial defect surgery procedure. (A) Shaving and skin preparation; (B) Disinfection; (C) Median incision along the sagittal midline, from the lateral canthus line to the posterior occipital area; (D) Exposure of the skull bone surface; (E) A 8mm dental trephine; (F) Preparation of cranial defect model; (G) Implantation the delivery system in the bony defect; (H) Suturing the periosteum and the skin.

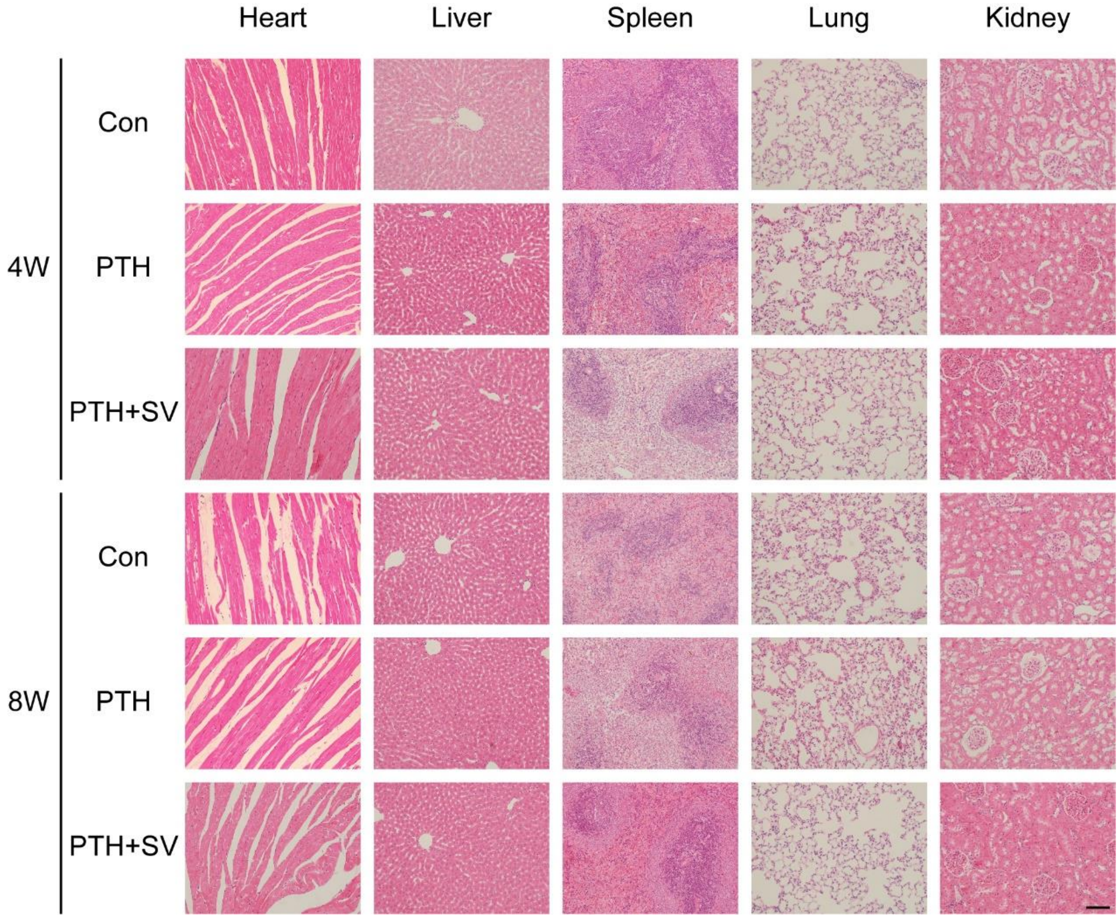

Supplementary Figure 12. HE-stained histological sections of the heart, liver, spleen, lung, and kidney. The scale bar indicates 100 μm. No obvious tissue damage was observed in all groups of organs after the composite system implantation at 4 weeks and 8 weeks, with normal morphological structure of cell nuclei, no obvious inflammatory cell infiltration, and no obvious pathological changes.

2 Supplementary Tables

Supplementary Table 1. Primer Sequence of MC3T3-E1 cells

Gene	Forward Sequence	Reverse Sequence
Bmp2	AAGCGTCAAGCCAAACACAAACAG	GAGGTGCCACGATCCAGTCATTC
Alp	CGGCGTCCATGAGCAGAACTAC	CAGGCACAGTGGTCAAGGTTGG
Ocn	CAGAGGAACTGGTTAGCAGGCAAC	ACGCAGGTTCTCAATGGCACAC
Col1	AGGGTCCCGCTGGTCAAGATG	ATGCCTGTTGCTGGTTCTGTAGTG
Gapdh	TGAACGGGAAGCTCACTGG	TCCACCACCCTGTTGCTGTA
